# Biomarkers of Allostatic Load as Correlates of Impairment in Youth with Chronic Pain: An Initial Investigation

**DOI:** 10.3390/children8080709

**Published:** 2021-08-18

**Authors:** Sarah Nelson, Samantha Bento, Michelle Bosquet Enlow

**Affiliations:** 1 Department of Anesthesiology, Critical Care and Pain Medicine, Boston Children’s Hospital, Boston, MA 02115, USA; samantha.bento@childrens.harvard.edu; 2 Department of Psychiatry, Harvard Medical School, Boston, MA 02115, USA; Michelle.Bosquet@childrens.harvard.edu; 3 Department of Psychiatry and Behavioral Sciences, Boston Children’s Hospital, Boston, MA 02115, USA

**Keywords:** pediatric pain, allostatic load, biomarkers, stress

## Abstract

Pediatric chronic pain is common and responsible for significant healthcare burden. However, the mechanisms underlying the development and/or maintenance of pediatric chronic pain remain poorly understood. Allostatic load (AL), or wear and tear on the nervous system following significant or prolonged stress, has been proposed to play a role in the maintenance of chronic pain, but minimal research has examined this possibility. This gap in research is particularly notable given the high exposure to adverse childhood experiences (ACEs; abuse/neglect, etc.) and psychological stress in this population. Accordingly, the current study aimed to preliminarily examine the measurement of AL in a treatment-seeking pediatric pain population. Biomarkers were collected during an already scheduled new patient pain evaluation and included salivary cortisol, dehydroepiandrosterone (DHEA), and C-reactive protein, in addition to waist–hip ratio, body-mass index, and blood pressure. A total of 61 children and adolescents with chronic pain (*M*age = 14.47 years; 88.5% female and white/Caucasian) completed study procedures and were included in analyses. Preliminary results indicated that a multifactorial AL composite is feasible to assess for in a tertiary pain treatment setting and that over 50% of youth with chronic pain were classified as high risk for AL (two or more risk factors). Further, it was found that individual AL risk factors were significantly associated with functional disability and that AL may moderate the association between psychosocial and functional outcomes. Given the pilot nature of this study, results should be used to inform future investigations with larger and more diverse pediatric pain samples.

## 1. Introduction

Chronic pain in youth is common, affecting 20–25% of youth [1], and is often associated with significant psychosocial (e.g., anxiety, depression) and pain-related (e.g., decreased physical activity, use of assistive devices) impairment [2,3]. The diagnosis and treatment of chronic pain in youth is highly burdensome on healthcare, imposing thousands of dollars of costs per person per year [4,5]. Thus, although pediatric pain engenders formidable individual and public health burdens, the mechanisms underlying the development and maintenance of chronic pain in youth remain understudied and poorly explicated. In particular, minimal examination of the neuroendocrine mechanisms behind pain and related outcomes has been performed in youth populations. One process that has been implicated in the initiation and/or maintenance of chronic pain in adults is allostatic load (AL) [6,7,8]. AL is a concept that encompasses significant “wear and tear” on the body’s sympathetic and parasympathetic nervous systems [9] and involves the body’s persistent release of catecholamines and glucocorticoids (e.g., cortisol) over time [10,11]. This phenomenon has been associated with a multitude of disease processes and is known to occur following repeated and/or prolonged exposure to stress [11,12], i.e., a negative emotional experience taking place concurrently with physiological, neuroendocrine, and/or behavioral changes [13,14]. Among other chronic health conditions, recent research indicates that AL accounts for increased nervous system sensitization and neuroendocrine dysfunction [6,10] and, thus, may play a causal role in the maintenance of chronic pain conditions in adults [6,8]. Parallel lines of research have outlined a stress model of chronic pain that implicates the HPA axis and glucocorticoid involvement in the maintenance of chronic pain conditions in adult populations [15]. A recent study provided more concrete evidence of this phenomenon in adults with fibromyalgia, who exhibited increased salivary cortisol levels compared to healthy controls [16]. However, these processes have not been examined in youth with chronic pain. This gap is particularly notable given evidence that youth with chronic pain experience high levels of stress [15,17] and that a majority (60–80%) have experienced at least one adverse childhood experience (ACEs; abuse, neglect, parent mental illness, parent divorce/separation, etc.) [18,19]. Historically, pediatric chronic pain populations have provided unique challenges for biomechanistic researchers due to their status as a vulnerable and highly sensitive population [20,21]. Examining neuroendocrine biomarkers of risk may be a particularly useful avenue for investigation and non-invasive method of assessment for these patient populations, but minimal research has examined this.

Irrespective of pain, AL has been found to be present and implicated in long-term health-related and psychosocial impairment in youth with a history of psychological trauma and/or ACEs [10,22]. It has been hypothesized that the experience of ACEs can maintain chronic pain and/or pain-related impairment in youth populations [8]. However, despite the high incidence rate of psychological trauma and/or ACEs in youth with chronic pain [18,23], there is a lack of research exploring the common mechanisms between these phenomena.

The current investigation is a pilot study that aimed to (1) examine the feasibility of a multifactorial AL composite, validated in other pediatric populations, in treatment-seeking youth with chronic pain, (2) examine associations between AL and pain-related and psychosocial impairment in youth with various chronic pain conditions, and (3) examine the magnitude of AL in youth with chronic pain and a history of ACEs relative to that of youth with chronic pain and no or minimal history of ACEs. We hypothesized that the assessment of a multifactorial AL composite is feasible to execute in a tertiary setting for youth with chronic pain. This hypothesis will be demonstrated by the collection of blood pressure, salivary samples to measure glucocorticoids (cortisol, dehydroepiandrosterone [DHEA]) and inflammation (c-reactive protein), waist/hip ratio (WHR), and body-mass index ([BMI] height and weight). Based on previous research and the high rate of ACEs documented in pediatric chronic pain populations, we also hypothesized that youth with chronic pain evidence elevated risk for AL when compared to previously published data and that youth with high AL risk experience increased pain-related and psychosocial distress compared to youth with chronic pain and low AL risk. Due to the pilot nature of this study and lack of extant research in this population, we explored both direct and indirect (i.e., moderation) associations between individual AL risk factors, the AL cumulative risk score, and outcomes.

## 2. Methods

### 2.1. Participant Sample

Participants for the current study (BCH IRB-P00027928; original approval date: 28 January 2018) included children and adolescents between the ages of 10–17 years scheduled for an initial evaluation in the pain treatment service (PTS) clinic at Boston Children’s Hospital (BCH; Boston, MA, USA). Eligible participants were patients with a chronic pain condition who were scheduled for a new outpatient PTS evaluation. Patients were excluded from recruitment if they were presenting for evaluation with chronic pain secondary to a specific disease-based process (e.g., sickle cell disease, juvenile-idiopathic arthritis), were not sufficiently fluent in English to complete questionnaires, and/or were severely cognitively impaired.

### 2.2. Recruitment Procedures

As part of a regularly scheduled new patient clinical evaluation, prospective participants were provided a study flier prior to their appointment detailing currently recruiting studies in the PTS. Potential participants were contacted by study staff via phone at least one week before their clinical appointment. During this call, they were provided with an overview of the study and presented with the option of participating. If they decided to participate, they were mailed a package containing a consent form, three saliva collection tubes (one for DHEA and two for cortisol), and written instructions for collecting and documenting the date and time of saliva sample collections. Participants were recontacted at least 48 h prior to their PTS appointment, at which time study staff completed consenting procedures with participants and their parent. During this call, participants were provided an overview of saliva collection procedures to be implemented on the morning of their PTS appointment. Specifically, participants were asked to record the time they woke up, then to immediately collect two saliva samples before brushing teeth or eating/drinking, and then, collect a third sample approximately 30 min later.

Upon presentation to BCH for their clinical appointment, participants were asked to provide their saliva samples and collection diary for labeling and storage. At this time, they were also provided with a tablet computer on which to access REDCap (Research Electronic Data Capture [24]) to complete additional questionnaires. They were escorted to a confidential exam room where their physiological outcome measures were recorded (i.e., blood pressure, WHR, BMI). Blood pressure, height, and weight were taken as part of standard vitals collection and performed by a Registered Nurse (RN). WHR ratio was obtained by a trained research coordinator. Participants were provided a USD $10 gift card for study participation.

### 2.3. Outcome Measures

#### 2.3.1. Demographic and Medical Information

Participants’ parents were asked to report the participant’s age, sex, and race/ethnicity. Participants and their parents were asked to report on the overall frequency and time since the first onset of the participant’s pain symptoms and on the general location or nature of their pain complaint.

#### 2.3.2. Allostatic Load (AL)

*Multifactorial AL Composite* [22,25] consists of the following:

HPA axis functioning: Cortisol and DHEA measures were assessed via three saliva samples collected using passive drool.

Inflammation: Inflammation was operationalized as C-reactive protein (CRP), which has proven to be a valid, generalized indicator of inflammation in pediatric samples [26]. CRP was measured from saliva collected using passive drool and garnered via secondary analyses following primary analysis of cortisol and DHEA.

Cardiovascular functioning: Blood pressure (BP) was measured by an RN during the clinical appointment.

Lipid functioning: Waist–hip ratio (WHR) was measured by a trained research coordinator and BMI by an RN at the clinical appointment.

#### 2.3.3. Pain-Related Functioning

*Pain Catastrophizing Scale (PCS-C)* [27] is a validated 13-item self-report measure of negative thinking related to pain with a cutoff of 26 (raw score) to be indicative of clinical significance. This version is based on the widely used adult PCS [28] and has been shown to be psychometrically sound for use with children ages 8–17. Participants completed the PCS-C as part of their clinical appointment.

*Fear of Pain Questionnaire (FOPQ-C)* [29] is a shortened version of the validated Pain Avoidance Questionnaire. It is self-report inventory to assess pain-related fears. Each item is rated on a 5-point Likert-type scale from 0 = “strongly disagree” to 4 = “strongly agree.” The FOPQ consists of 24 items with strong internal consistency, validity, and reliability. Cutoff scores are as follows: 0–34—low fear; 51–96—high fear. This measure is validated for children ages 8–17 [29]. Participants completed this as part of their clinical appointment.

*Numeric Rating Scale (NRS) Pain Intensity—Child Report* [30] assesses “usual” (i.e., average or typical), “best” (i.e., lowest), and “worst” (i.e., highest) pain intensity (0–10 scale) over the last two weeks, with “0” indicating no pain and “10” reflecting worst possible pain. The NRS pain intensity scale is recommended for use in clinical studies of pain in school-age children [31].

*Functional Disability Inventory (FDI)* [31,32] is a self-report measure of perceived difficulty in performing activities in school, home, physical, and social contexts. The FDI is widely used in pediatric pain research and is recommended as the gold-standard measure of physical functioning for school age children and adolescents for clinical trials in pediatric chronic pain [31]. It has been validated for children ages 8–17. Cutoff scores are as follows: 0–12—no/minimal disability; 13–29—moderate disability; 30+—severe disability. Participants completed this as part of their regularly scheduled clinical appointment.

#### 2.3.4. Psychosocial Functioning

*PROMIS Pediatric Short Forms* [33] are validated self-report questionnaires that examine a variety of aspects related to child/adolescent physical, mental, and social health. Each measure was developed to be a unidimensional assessment of the construct using only the most informative items. The response format is consistent across measures and is based on a 5-point Likert scale with the anchors “never” (0) and “almost always” (4). Raw score totals on each measure can be converted to T-scores with higher scores indicative of greater impairment. For the current study, the Sleep Disturbance (8 items), Anxiety (8 items), Depressive Symptoms (8 items), and Psychological Stress (8 items) pediatric self-report short forms (ages 8–17) were administered during the clinical appointment.

#### 2.3.5. Exposure to Stressful Experiences

*Childhood Trust Events Survey (CTES)* [34] is a questionnaire designed to screen for a variety of lifetime stressful life events, including adverse childhood experiences (ACEs), in youth. Items are scored as yes/no for the experience of an event and summed for a total exposure score, with higher scores indicating greater exposure to stressful life events. For the purposes of the current study, only the parent-report version was administered, and only events that were consistent with the original ACEs categorization were used [35].

### 2.4. Analyses

#### 2.4.1. Power Analysis

Power analyses were performed using GPower [36,37]. Based on prior research in both pain and ACEs studies [23,38], we determined that a total sample size of 60 would provide excellent (80% or higher) statistical power to detect moderate correlations for within group analyses between the multifactorial AL composite and identified outcomes (i.e., pain intensity, physical and psychosocial impairment) based on two-tailed alpha of 0.05.

#### 2.4.2. Saliva Assay

All saliva samples were frozen and stored at −80 °C until analysis. For cortisol and DHEA, samples were shipped on dry ice to Dresden Lab Service (Dresden, Germany). After thawing, salivettes were centrifuged at 3000 rpm for 5 min, which resulted in a clear supernatant of low viscosity. Salivary concentrations were measured using commercially available chemiluminescence immunoassays with high sensitivity (IBL International, Hamburg, Germany). The intra- and inter-assay coefficients for cortisol and DHEA were below 8% and 10%, respectively.

For CRP analyses, samples that has been analyzed for cortisol and/or DHEA were shipped on dry ice to the Stress Physiology Investigative Team (SPIT) lab (Iowa State University, Ames, IA, USA) for enzyme immunoassays. Each saliva sample was thawed, centrifuged for 15 min at 3000 rotations per minute, and assayed within 8 h in duplicate using well-established enzyme immunoassay kits (salimetrics). The mean intra-assay coefficient of variation (CV) was 11.6% and mean inter-assay CV was 2%. Samples were reanalyzed if the CV for the duplicate measurements was greater than 10%, based on optical density. To normalize distributions across all assay results, extreme values were winsorized to 2 SDs of the median, and raw values were log-transformed. The number one was added to all log-transformed cortisol values to address negative values.

#### 2.4.3. Allostatic Load Coding

BP risk was coded using a standardized measurement system by a computer software program (EZ BP calculator) [39] and conceptualized by the software as prehypertension, stage 1 hypertension, and stage 2 hypertension. Risk in the areas of WHR, BMI, CRP, and DHEA was all coded using the 75th percentile of the sample as metric to capture those participants with the highest scores in each area. Specifically, each score at or above the 75th percentile was assigned a 1 and added to the total AL score. This method has been used successfully in other AL risk models [40]. For cortisol AL risk, cortisol rise (“morning cortisol”) was coded using the change in scores (Sample 2–Sample 1) between baseline cortisol collected at awakening (Sample 1) and the sample collected 30 min after awakening (Sample 2). Participants were assigned a “1” on the risk rating if their change in cortisol between timepoints was in the bottom 25th percentile of the sample (meaning little change or decline in values), which is consistent with previous research [22,40]. Due to the pilot nature of the current study, the relation between individual and the sum of AL risk factors and pain-related and psychosocial outcomes will be examined.

#### 2.4.4. Data Analyses

All analyses were performed using SPSS v. 26 [41]. Feasibility of the current study procedures was measured using participant and clinician reporting (see below) and measures of frequency in participant recruitment and completion. Participants were excluded from analyses if they were missing all saliva analyses but were included if only one of three samples were missing. Next, following individual coding of AL risk factors, an AL composite risk score was formulated by combining each individual factor for a total composite, with possible scores ranging from 0 to 6. Additionally, these data were recoded dichotomously to capture those with high AL risk, as indicated by the presence of two or more (coded as 1 = high AL risk) versus fewer than two (coded as 0 = low AL risk) factors. Similarly, an ACEs variable was coded continuously (i.e., total exposure count) and categorically (i.e., 0, 1, or 2 or more ACEs), in line with previous research [18,42]. Using these variables, measures of central tendency and variability were performed to report trends in the sample and across AL risk score groupings. To account for the small sample size, non-parametric analyses were used.

Spearman’s correlations were used to examine (1) associations among individual AL variables, the overall AL risk score, and ACE variables and (2) the association between individual AL risk variables (continuously coded) and study outcomes, including pain intensity (“best,” “worst,” “typical”), functional disability, anxiety and depressive symptoms, psychological stress, fear of pain, and pain catastrophizing. The cortisol: DHEA ratio was calculated, and correlation analyses were conducted to examine the relation between this ratio and study outcomes, as identified above. Next, a series of Mann–Whitney U tests were performed with the individual dichotomously coded AL risk variables as the grouping variable and study outcomes as the dependent variables. Finally, in order to examine AL risk status (high vs. low) as a moderator of impairment, the sample was split by AL risk (0 vs. 1), and correlations within the subsamples were tested separately. In each set of analyses, Spearman’s correlations were performed among all relevant study outcomes, and then, Fisher r-to-z transformations were run to examine group differences.

## 3. Results

### 3.1. Sample Characteristics

Sixty-two children and adolescents with chronic pain consented for enrollment into the study; one participant was excluded from the current analyses due to non-completion of any saliva procedures for an analytic sample of N = 61 (*M*age = 14.47 years). Participants were predominantly female (88.5%) and white/Caucasian (88.5%), which is consistent with the demographics of pediatric pain samples [1,43]. The most common pain complaint in the current sample was widespread or multiple pain locations (e.g., fibromyalgia—34.4%), with the next most common being complex regional pain syndrome (CRPS) or amplified musculoskeletal pain syndrome (AMPS). Among participants, descriptive analyses indicated that 70.5% endorsed at least one adverse childhood experience (by formal definition [35]) with almost half of the sample (45.9%) endorsing two or more ACEs (*M_ACE_* = 1.8, SD = 1.76). The sample represented a range of impairment in psychosocial functioning (anxiety symptoms *M* = 50.76, SD = 10.67; depressive symptoms *M* = 52.71, SD = 9.93), psychological stress (*M* = 56.96, SD = 9.28), and sleep disturbances (*M* = 60.14, SD = 7.46). Sample mean scores were in the moderate range for functional disability (*M* = 21.82, SD = 11.78) and usual pain intensity (*M* = 6.4, SD = 1.9) ratings. Full descriptive results of demographic and key outcome measures are detailed in Table 1.

### 3.2. Feasibility of AL Measurement in Tertiary Care

Results of this pilot study indicated that a multifactorial AL composite is feasible to collect within a tertiary chronic pain clinic. When presented in the context of an already scheduled medical visit, recruitment rates for pain patients who were directly spoken to (vs. leaving a voicemail) were 93%. Data collection challenges included not being able to directly connect with patients prior to their medical appointment to present them with the option of study participation and participants not remembering to bring their completed saliva samples to their clinical appointment. Out of all enrolled participants, 92% completed all study procedures in conjunction with their clinical evaluation. One failed to complete any saliva procedures (i.e., did not bring completed saliva samples on the morning of the appointment) and was, thus, excluded from the current analyses. Four participants brought in two of three completed samples (AL risk factor coded as missing). Vital measurements (i.e., BP, WHR, weight/height) and an ACEs screener via tablet were successfully gathered from all participants. Study procedures required 15–20 min of the participant’s time above that of the clinical appointment and did not interfere with clinic proceedings, per healthcare providers’ anecdotal report.

### 3.3. Frequency of AL Risk Factors in Youth with Chronic Pain

A large majority of participants (80.3%) evidenced at least one risk factor for AL, with 57.4% of participants with two or more risk factors—conceptualized as “high risk for AL” [44]. Across participants, the AL composite risk score ranged from 0 to 6 (*M*score = 1.7; SD = 1.50). Full descriptive statistics on individual AL risk factors are detailed in Table 1.

Spearman’s correlations were performed to examine the associations between individual AL risk factors (continuous log-transformed variables) and the cumulative AL risk score. Correlation coefficients ranged from *r_s_* = 0.277 to *r_s_* = 0.490. When combined into one correlation model, the total AL ratio was most robustly associated with BMI (*r_s_* = 0.490, *p* < 0.001) and morning cortisol (*r_s_* = 0.485, *p* < 0.001). The weakest correlations were with WHR (*r_s_* = 0.366, *p* = 0.004) and CRP (*r_s_* = 0.277, *p* = 0.042). The continuous ACEs variable was not associated with the categorical AL risk score or with any of the individual risk factors except for WHR (*r_s_* = −0.374, *p* = 0.003).

### 3.4. Association(s) between AL Risk and Pain-Related and Psychosocial Outcomes

Results of individual Spearman’s correlation analyses revealed associations between BMI and FDI (*r_s_* = −0.395, *p* = 0.002) and between DHEA and “best pain” (*r_s_* = −0.326, *p* = 0.011), which (separately) indicates that as BMI and DHEA increase, functional disability and “best pain” scores decrease. Results were trending towards significance for the associations of BMI with psychological stress (*r_s_* = −0.252, *p* = 0.05) and fear of pain (*r_s_* = −0.237, *p* = 0.076) and of DHEA with FDI (*r_s_* = −0.244, *p* = 0.069).

Results of Mann–Whitney U tests with bivariate risk factors as the grouping variable and identified outcomes as the dependent variables revealed differences across functional disability by DHEA risk grouping (i.e., <75 percentile vs. ≥75 percentile—z = −2.011, *p* = 0.044), with results indicating that the low DHEA group generally reported higher functional disability (*n* = 43; mean rank = 30.91) than the high DHEA group (*n* = 13; mean rank = 20.54). No other differences by risk group were revealed for any outcome. The difference in functional disability by morning cortisol risk was trending towards significance (z = −1.724, *p* = 0.085), with data trends indicating that the lower cortisol group tended to report higher functional disability (*n* = 45; mean rank = 30.96) than the high cortisol group (*n* = 15; mean rank = 21.67).

Descriptive data for the cortisol and DHEA variables as well as the cortisol: DHEA ratio are presented in Table 1. Results were trending towards significance for the associations between the cortisol: DHEA ratio and functional disability (*r_s_* = 0.244, *p* = 0.069). 

Cumulative AL Risk Score: Consistent with previous studies, participants were coded as high risk for AL if they evidenced two or more AL risk factors [44]. Table 2 details the distribution of key demographic and outcome variables by AL risk status (>2 or ≤2). In order to examine AL risk as a moderator of impairment, the sample was split by AL risk status (0, 1), and bivariate Spearman’s correlations were run across all study outcome variables. Correlations are provided in Table 3. Within the high-risk AL group but not within the low-risk AL group, the associations among sleep, depression, pain catastrophizing, psychological stress, and fear of pain were significant. Similarly, significant correlations were found among depressive symptoms and “best pain” ratings in the low- but not in the high-risk AL group. Using the Fisher r-to-z transformation, significant moderation was found, as it was revealed that anxiety (z = −2.1, *p* = 0.036) and psychological stress (z = −2, *p* = 0.045) were more strongly associated with sleep disturbances in the high-risk vs. low-risk AL group. Results were trending in the same direction for depressive symptoms and best pain (z = 1.96, *p* = 0.05) and sleep and fear of pain (z = −1.77, *p* = 0.076). Conversely, results were nearing significance in the opposite direction by indicating that a history of ACEs were more strongly associated with sleep (z = 1.73, *p* = 0.08) and anxiety (z = 1.78, *p* = 0.075) in the low-risk AL vs. high-risk AL groups.

## 4. Discussion

Chronic pain is common in youth, but the mechanisms underlying its initiation and maintenance are poorly understood. One potential mechanism, which has been implicated in chronic pain maintenance, is allostatic load (AL), a multifactorial construct that represents the physiological “wear and tear” that prolonged and/or intensive stress can have on the body [6,10,11]. However, this construct has not received attention in studies of youth with chronic pain, despite the high incidence of ACEs and psychological stress observed in pediatric pain populations [17,18,19,23,45].

In the current study, a multifactorial AL model was developed and examined in relation to pain and pain-related disability. The individual AL factors included measures of HPA-axis functioning (i.e., morning cortisol, cortisol rise, DHEA [22,25]), cardiovascular functioning (i.e., blood pressure [25]), inflammation (i.e., C-reactive protein [25,44]), and lipid functioning (waist–hip ratio, body-mass index [22,25]). Results from this pilot study, implemented within a tertiary pediatric chronic pain clinic, indicate that this model is feasible to carry out, does not interfere with normal clinic functioning, and is acceptable for patients who are presenting for specialty care evaluation. We found that approximately 80% of patients evidenced at least one risk factor for AL, with over 50% having two or more AL risk factors, which has been labeled as “high AL risk” in previous studies [44]. These findings are comparable albeit higher than the 35% documented in a nationally representative sample of adolescents [44].

Among individual AL risk factors, results of correlational analyses indicated that BMI and morning cortisol levels had the strongest associations with overall AL risk. Individually, BMI has been associated with ACEs [46,47] and with pain in youth [48]. Future research should consider BMI, stress, and pain jointly to better understand the pain-related risk and trajectory of long-term impairment in youth who have a history of stress and at-risk BMI status. With respect to cortisol, data trends evidenced an association between a flattened morning cortisol response and higher functional disability. In general, a blunted or flattened cortisol pattern, in contrast to the expected morning rise, is indicative of an altered ability of the HPA axis to adaptively respond to stress and daily demands, a process important for overall health [49,50,51]. Diurnal salivary cortisol has been associated with increased pain in adult populations [52]. This relation was not found in the current sample, suggesting that childhood may be a unique time to address the potential role of cortisol in pain maintenance as a strategy for long-term prevention of suffering.

Although the association between DHEA and the overall AL risk score was relatively modest, we found that youth with higher levels of DHEA evidenced lower levels of “best pain.” In previous research, DHEA has been deemed to be “neuroprotective,” [53] potentially acting as a buffer against the potential impact of “neurotoxic” cortisol levels [53] and other AL risk factors. Consistent with these findings, exploration of the cortisol: DHEA ratio revealed trending associations with respect to functional disability, with higher ratios associated with increased functional disability. These results suggest that DHEA, either alone or in combination with a cortisol/DHEA ratio, may be a valuable avenue for future investigation to identify mechanisms of resiliency and treatment optimization for these youth.

Analyses failed to reveal differences in impairment by AL risk group, suggesting that AL risk factors may not directly and/or solely influence pain. However, the findings also suggested that AL risk may *moderate* associations among anxiety, psychological stress, and sleep impairment in youth with chronic pain, given that those in the high AL but not in the low AL risk grouping evidenced associations among these outcomes. These findings are important to consider in the context of previous literature, as the association between psychological functioning and sleep in pediatric pain populations has been well established [54,55,56]. It may be that targeting AL in these youth via appropriate interventions could buffer the impact of psychological distress on sleep and, in turn, on pain, but these relations have yet to be examined. Across interventions, specifically examining AL risk factors (i.e., objective measures) vs. psychosocial outcomes (i.e., subjective measures) as intervention outcome(s) could also provide more effective understanding of the role that these mechanisms may play in impairment. Future research should further examine this.

Given the pilot nature of the current study, it may be worth considering results trending toward significance in order to inform future investigations. Specifically, the associations between depression and “best pain” and between fear of pain and sleep were trending toward significance in the high AL group only. These data trends may support considering AL when conceptualizing the already well-established [57,58,59] relation(s) between psychosocial [10,12] and health-related [55,60] behaviors in pediatric pain samples. Interestingly, associations approached significance between ACE history and sleep and anxiety in the low AL risk grouping only. It may be that poor health-related behaviors and general levels of stress contribute more to AL risk status than specific ACEs, which is contrary to what has been hypothesized previously. This may be an artifact of the demographics of treatment-seeking pediatric pain samples, which are frequently of higher socioeconomic status than community-based samples [61]. Future research should examine these potential differences.

The current study has several strengths, including the recruitment of a representative pediatric pain sample, which lends towards increased generalizability of preliminary findings to other treatment-seeking youth. The use of empirically validated questionnaires and objective measures of physiological functioning (e.g., saliva sampling) likely also enhanced the validity of results. Saliva assay may be a particularly useful method for measuring neuroendocrine functioning vs. blood analysis given the increased pain involved with an already sensitive and vulnerable patient population (i.e., pediatric chronic pain). Future research should continue to examine the validity of this methodology. Important limitations in the current study should also be acknowledged. First, the current study revealed several non-significant results, despite adequate power. This may be partially explained by pilot study methods that can be optimized in future investigations (e.g., inclusion of control group, larger sample size for more defined between vs. within subject’s analyses, etc.). Further, the cortisol findings are limited by the collection of only two samples, which is not fully representative of the functioning of the HPA axis. Additionally, prior research has identified several other potential indicators of AL that were not considered in the current study, including epinephrine/norepinephrine, cholesterol, high density lipoprotein, and resting heart rate [11,25], thus limiting the scope of the AL composite risk score. The use of a treatment-seeking sample limits the generalizability of the findings to the general adolescent population. This study methodology also did not include current medications in the interpretation of the saliva assay, which is also a limitation and should be examined in future research. Finally, reliance on self- or other reporting (youth or parent) of symptoms is a limitation, as symptom reporting may not be fully indicative of the patient’s clinical picture.

Results of the current study may be used as a foundation for several important avenues of future research. To begin to understand more fully the manifestation of AL in pediatric pain participants, the measurement of AL should be repeated in larger and more diverse samples with the inclusion of a greater variety of AL biomarkers (e.g., resting heart rate, epinephrine/norepinephrine, etc.). Assessing for AL and pain-related symptoms in nationally representative samples of youth may also aid in the understanding of AL as a potential risk factor for long-term pain-related impairment. Finally, given that high AL risk was identified as a potential moderator of impairment and has been shown to be positively responsive to treatment [62], using AL as a target of intervention via pharmacological or non-pharmacological methods (e.g., physical therapy, psychological therapies) may be an important avenue for optimizing long-term treatment responses in these youth.

## Figures and Tables

**Table 1:** Sample Characteristics; N = 61.

	M (SD)	Range
Age	14.47 (1.96)	10–17
Gender (*n*, %)		
Female	54 (88.5)	
Male	6 (9.8)	
Other	1 (1.6)	
Race/ethnicity (*n*, %)		
White	54 (88.5)	
Black	3 (4.9)	
Asian	1 (1.6)	
Hispanic or Latino	1 (1.6)	
American Indian or Alaskan Native	1 (1.6)	
Other	2 (3.3)	
Pain location(s) * (*n*, %)		
Widespread/multiple pains	21 (34.4)	
CRPS/AMPS	18 (29.5)	
Back/chest	9 (14.8)	
Other	13 (21.3)	
Cortisol	8.24 (5.1)	0.15–18.10
DHEA	315.82 (212.35)	18.62–740.05
Cortisol: DHEA ratio	0.05 (0.09)	0.00–0.66
C-reactive protein (CRP)	294.76 (413.04)	2.42–1128.79
Waist/hip ratio (WHR)	0.78 (0.06)	0.60–0.91
Body-mass index (BMI)	22.71 (5.56)	14.60–22.71
PCS-C	25.74 (12.61)	4–47
FOPQ-C	43.51 (20.32)	0–79
Pain intensity		
Usual pain	6.43 (1.87)	0–10
Worst pain	8.04 (1.47)	4–10
Best pain	4.65 (2.55)	0–10
FDI	21.82 (11.78)	0–50
PROMIS measures		
Sleep disturbance	60.14 (7.46)	46.8–78.5
Anxiety	50.76 (10.67)	33.5–74.6
Depression	52.72 (9.94)	35.2–71.4
Psychological stress	56.96 (9.28)	37–81.8
CTES	1.51 (1.68)	0–6
None (*n*, %)	18 (29.5)	
1 ACE (*n*, %)	15 (24.6)	
2 ACEs (*n*, %)	10 (16.4)	
≥3 ACEs (*n*, %)	18 (29.5)	

Abbreviations: PCS-C, Pain Catastrophizing Scale (child version); FOPQ-C, Fear of Pain (child version); FDI, Functional Disability Inventory; CTES, Childhood Trust Events Survey; ACE, adverse childhood event. Variables are presented as mean (standard deviation) with their respective ranges, or *n* (%). * Pain locations: CRPS, complex regional pain syndrome; AMPS, amplified musculoskeletal pain syndrome; Other, pelvic pain, localized pain without diagnosis, etc.

**Table 2:** Distribution of Key Demographic and Outcomes Variables across AL Risk Status.

	AL (<2); *n* = 26	AL (≥2); *n* = 35
Age	14.65 (2.19)	14.32 (1.79)
Gender (*n*, %)		
Female	24 (92.3)	30 (85.7)
Male	1 (3.8)	5 (14.3)
Other	1 (3.8)	0 (0)
Race/ethnicity (*n*, %)		
White	24 (92.3)	30 (85.7)
Black	1 (3.8)	2 (5.7)
Asian	1 (3.8)	0 (0)
Hispanic or Latino	0 (0)	1 (2.9)
American Indian or Alaskan Native	0 (0)	1 (2.9)
Other	2 (19.2)	2 (5.7)
Frequency of pain (*n*, %)		
A couple of times per year	0 (0)	2 (5.7)
Once a month	0 (0)	1 (2.9)
Everyday	6 (23.1)	8 (22.9)
Other	20 (76.9)	24 (68.6)
Cortisol	7.24 (4.37)	8.99 (5.53)
DHEA	224.75 (176.51)	380.84 (214.03)
Cortisol: DHEA ratio	0.05 (0.05)	0.05 (0.11)
C-reactive protein	367.53 (470.02)	240.78 (363.76)
Waist/hip ratio (WHR)	0.75 (0.03)	0.79 (0.07)
Body-mass index (BMI)	20.62 (2.70)	24.26 (6.58)
PCS-C	24.21 (13.13)	26.85 (12.30)
FOPQ-C	42.04 (19.46)	44.58 (21.15)
Pain intensity		
Usual pain	6.47 (1.61)	6.41 (2.05)
Worst pain	8.24 (1.21)	7.89 (1.64)
Best pain	4.49 (2.56)	4.77 (2.57)
FDI	23.0 (10.50)	20.97 (12.72)
PROMIS measures		
Sleep disturbance	59.18 (6.55)	60.92 (8.14)
Anxiety	50.93 (10.15)	50.60 (11.19)
Depression	53.33 (9.87)	52.27 (10.11)
Psychological stress	57.1 (8.60)	56.85 (9.88)
CTES		
None (*n*, %)	4 (15.4)	14 (40)
1 ACE (*n*, %)	5 (19.2)	10 (28.6)
2 ACEs (*n*, %)	6 (23.1)	4 (11.4)
≥3 ACEs (*n*, %)	11 (42.3)	7 (20)

Abbreviations: PCS-C, Pain Catastrophizing Scale (child version); FOPQ-C, Fear of Pain (child version); FDI, Functional Disability Inventory; PROMIS, Patient-Reported Outcomes Measurement Information System; CTES, Childhood Trust Events Survey; ACE, adverse childhood event. Variables are presented as mean (standard deviation) or *n* (%).

**Table 3:** Bivariate Spearman’s Correlation Coefficients across Study Outcomes by AL Risk Status (≤2: Unshaded; >2: Shaded).

	1	2	3	4	5	6	7	8	9	10
1. PCS	1	0.79 **	0.50 **	0.48 **	0.34	0.28	0.41 *	0.36 *	0.26 *	0.12
2. FOPQ-C	0.75 **	1	0.70 **	0.59 **	0.50 **	0.53 **	0.57 **	0.57 **	0.45 **	0.25
3. FDI	0.50 *	0.67 **	1	0.56 **	0.39 *	0.26	0.31	0.68 **	0.48 **	0.47 **
4. PROMIS Sleep	0.07	0.27	0.36	1	0.72 **	0.60 **	0.64 **	0.16	0.23	0.15
5. PROMIS Anxiety	0.66 **	0.63 **	0.51	0.44 *	1	0.76 **	0.75 **	0.15	0.21	0.11
6. PROMIS Depression	0.62 **	0.68 **	0.45 *	0.12	0.64 **	1	0.87 **	0.02	0.05	−0.03
7. PROMIS Stress	0.66 **	0.63 **	0.33	0.33	0.73 **	0.73 **	1	0.08	0.15	−0.02
8. Usual pain	0.16	0.04	0.19	−0.03	−0.15	−0.25	−0.26	1	0.75 **	0.57 **
9. Worst pain	0.18	0.18	0.03	−0.04	−0.12	−0.12	−0.09	0.60 **	1	0.35 *
10. Best pain	−0.02	−0.16	0.06	0.00	−0.46	−0.46 *	−0.38	0.49 *	0.12	1

Note. * Correlation coefficient is significant at the 0.05 level (2-tailed). ** Correlation coefficient is significant at the 0.01 level (2-tailed). PCS-C, Pain Catastrophizing Scale (child version); FOPQ-C, Fear of Pain (child version); FDI, Functional Disability Inventory; PROMIS, Patient-Reported Outcomes Measurement Information System.

## Data Availability

The data presented in this study are available on request from corresponding author. The data are not publicly available due to restrictions.
